# Anti-Microbial, Anti-Oxidant, and α-Amylase Inhibitory Activity of Traditionally-Used Medicinal Herbs: A Comparative Analyses of Pharmacology, and Phytoconstituents of Regional Halophytic Plants’ Diaspora

**DOI:** 10.3390/molecules25225457

**Published:** 2020-11-20

**Authors:** Mohsen S. Al-Omar, Hamdoon A. Mohammed, Salman A. A. Mohammed, Essam Abd-Elmoniem, Yasser I. Kandil, Hussein M. Eldeeb, Sridevi Chigurupati, Ghassan M. Sulaiman, Hadeel K. Al-Khurayyif, Basma S. Almansour, Prarthana M. Suryavamshi, Riaz A. Khan

**Affiliations:** 1 Department of Medicinal Chemistry and Pharmacognosy, College of Pharmacy, Qassim University, Qassim 51452, Saudi Arabia; m.omar@qu.edu.sa (M.S.A.-O.); ham.mohammed@qu.edu.sa (H.A.M.); S.Chigurupati@qu.edu.sa (S.C.); BS.Almansour@qu.edu.sa (B.S.A.); 2 Medicinal Chemistry and Pharmacognosy Department, Faculty of Pharmacy, JUST, Irbid 22110, Jordan; 3 Department of Pharmacognosy, Faculty of Pharmacy, Al-Azhar University, Cairo 11371, Egypt; 4 Department of Pharmacology and Toxicology, College of Pharmacy, Qassim University, Qassim 51452, Saudi Arabia; salmanafroze1@gmail.com (S.A.A.M.); husseineldeeb@qumed.edu.sa (H.M.E.); 5 Department of Plant Production and Protection, College of Agriculture and Veterinary Medicine, Qassim University, Qassim 51452, Saudi Arabia; ruessam2@yahoo.com; 6 Biochemistry and Molecular Biology Department, Faculty of Pharmacy, Al-Azhar University, Nasr City, Cairo 11231, Egypt; Y.Kandil@ammanu.edu.jo; 7 Pharmacological and Diagnostic Research Centre, Faculty of Pharmacy, Al-Ahliyya Amman University, Amman 19328, Jordan; 8 Department of Biochemistry, Faculty of Medicine, Al-Azhar University, Assiut 71524, Egypt; 9 Division of Biotechnology, Department of Applied Sciences, University of Technology, Baghdad 35010, Iraq; 100135@uotechnology.edu.iq; 10 College of Pharmacy, Qassim University, Qassim 51452, Saudi Arabia; had131141@gmail.com; 11 Department of Medical Laboratory, College of Applied Medical Sciences, Qassim University, Qassim 51452, Saudi Arabia; pm.suryavamshi@qu.edu.sa

**Keywords:** total phenolics, flavonoids, phenols, trace elements, anti-oxidant, anti-microbial, α-amylase enzyme inhibition, anti-diabetics, halophytes, *Aeluropus lagopoides*, *Salsola cyclophylla*, *Salsola imbricata*, *Tamarix aphylla*, *Zygophyllum simplex*, methicillin resistance *Staphylococcus aureus* (MRSA)

## Abstract

Halophytes are the category of plants growing under harsh conditions of super-salinity, and are wide-spread in the coastal Mediterranean climatic conditions and desert oasis. They are adept at surviving through maintaining excessive production of enzymatic, and non-enzymatic secondary metabolites, especially phenolics and flavonoids that primarily work as anti-oxidants and phytoalexins. Five major halophyte species growing in the kingdom’s Qassim’s high-salted desert regions were investigated for confirming their traditionally used biological activity of sugar-control and anti-infectious properties. In this context, the comparative presence of phenolics, and flavonoids together with anti-microbial, anti-oxidants, and the anti-diabetic potentials of the plants’ extracts were investigated through the α-amylase inhibition method. The highest concentrations of phenolics and flavonoids were detected in *Salsola imbricata* (360 mg/g of the extract as Gallic-Acid-Equivalents/GAE, and 70.5 mg/g of the extract as Rutin-Equivalents/RE). In contrast, the lowest concentrations of phenolics and flavonoids were detected in *Salsola cyclophylla* (126.6 mg/g GAE, and 20.5 mg/g RE). The halophytes were found rich in trace elements, a factor for water-retention in high-salinity plants, wherein iron and zinc elements were found comparatively in higher concentrations in *Aeluropus lagopoides* (4113 µg/kg, and 40.1 µg/kg, respectively), while the copper was detected in higher concentration (11.1 µg/kg) in *S. imbricata*, analyzed through Inductively Coupled Plasma Optical Emission Spectrometric (ICP-OES) analysis. The anti-oxidant potentials and α-amylase enzyme inhibition-based anti-diabetic activity of *S. imbricata* was significantly higher than the other halophytes under study, wherein *S. cyclophylla* exhibited the lowest level of α-amylase inhibition. The maximum DPPH radicals’ (52.47 mg/mL), and α-amylase inhibitions (IC_50_ 22.98 µg/mL) were detected in *A.* *lagopoides*. The anti-microbial activity against the Methicillin-Resistant *Staphylococcus aureus* was strongly exhibited by *Zygophyllum simplex* (33 mm Inhibition Zone-Diameter, 50 µg/mL Minimum-Inhibitory-Concentration), while *Escherichia coli*, *Enterococcus faecalis*, and *Candida albicans* growths were moderately inhibited by *Tamarix aphylla*. The current findings exhibited significant differences among the locally distributed halophytic plants species with regards to their bioactivity levels, anti-oxidant potentials, and the presence of trace elements. The ongoing data corroborated the plants’ traditional uses in infections and diabetic conditions. The enhanced local distribution of the plants’ diaspora and higher density of occurrence of these plants species in this region, in comparison to their normal climatic condition’s counterparts, seemed to be affected by humans’ use of the species as part of the traditional and alternative medicine over a period of long time.

## 1. Introduction

The halophytes are a group of plants that have the distinctive capability to survive and sustain in the harsh dry and high-salt-soil conditions [1]. These plants are widely distributed in the regions of the world wherein super-salinity environments exist with moderate to high temperatures together with a certain degree of moisture [2]. In the Kingdom of Saudi Arabia, the halophytic plants are commonly growing, some in high abundance, some in the coastal, and the central warm with low-rainfall, and high-salt soil locations [3,4]. The majority of the halophytes are traditionally used by the locals, and nomadic Bedouin tribes as a remedy for infectious, diabetic, and adverse skin conditions [5,6,7,8]. The human indulgence has widely promoted the distribution of these species, and their enhanced occurrence density in the region. Besides, these halophytes also form the part of the coveted grazing pastures of the sparsely distributed herbs which are used as livestock-feeds, and as an appetizer for camels in the central desert regions of Saudi Arabia [4].

The halophytes produce primary and secondary metabolites that have the potential to overcome the intracellular oxidative stress caused by salinity environments [9,10]. Moreover, these plants have the potential to be a supplemental source for natural anti-oxidants as well as certain trace elements. Their potential to be a part of a plant-based nutraceuticals source is imminent. Besides, the majority of the halophytes have traditionally been used, locally and worldwide, as a remedy for many diseases, and several symptomatic disorders, e.g., diabetes, hepatitis, jaundice, inflammation, and hypertension, have been treated or claimed to be treated. Nonetheless, the halophytes have also been used for the treatment of viral and bacterial infections and fevers as well [11]. The concurrently enhanced outlook and increased demands for complementary and alternative medicines in solving several therapeutic challenges, including the microbial resistance dilemma for regular and over-prescribed antibiotics, are concomitant. The indigenous systems of medicines have also propelled the search for natural anti-microbial molecular templates, new chemical entities, and leads for developing newer anti-microbial drug candidates, thereby initial screenings of plants of diversified environments have caught specific attention [12,13]. Besides, the ongoing search to find new structural templates, and novel molecular entities-based remedies from the natural products reservoir and pools of the plants’ kingdom for non-communicable diseases, e.g., diabetes, hypertension, neurodegenerative diseases, and diseases of the central nervous system, is rapidly taking shape worldwide [14,15]. The halophytes growing under duress are, supposedly, a rich and renewable source of biologically active secondary metabolites, hence upon demonstrating their roles in diversified biological activities evaluations [16]. These plants are also normally used as part of traditional herbal remedies with every indigenous system of medicine and are of high folk-lore reputation in the parts of the world they perennially survive. In this region, the locals, and nomadic people, frequently use these plants as a remedy for certain physiological conditions, hormonal disorders based on symptomatic observations, and the diseases identified through primitive diagnosis, as well as in wounds, burns, cuts, bad skin conditions, pains, swellings, and joint-swellings [17,18,19,20,21]. These plants are also a rich source of alternate nutrition for animals as well as a livestock-feed at times as a substitute for regular but standby green forage, the berseem, *Trifolium alexandrinum*.

As part of the plant wealth resources and traditional herbal medicament, the halophytes have a strong presence in the Mediterranean regions and Peninsular Arabia. These plant species also grow in the normal climatic zones, as well as have a higher occurrence in the salty environment owing to their better adaptation capabilities. Among the major halophytes of the region, the *Salsola imbricata*, and *Salsola cyclophylla*, which are also part of camels’ feed, besides being the commonest remedy for diarrhea, is also used against worms’ infestation, stomach ache, and dysentery [22]. The *Tamarix aphylla* and *Aeluropus lagopoides* are employed for wound healing, anti-diabetic, and also as pain-remover [23], whereas the *Zygophyllum simplex* is commonly used in the treatments of various diabetic conditions, wound-healing, and as an analgesic [24]. However, there is no significant work reported on the phytochemical constituents and biological activity of these major halophytes of the Saudi Arabian regions. The five of the widely, and wildly growing halophytes, *A. lagopoides*, *S. imbricata, S. cyclophylla*, *T. aphylla*, and *Z. simplex*, of central Saudi Arabia, have been investigated for their in vitro anti-microbial, anti-oxidant, and anti-diabetic activities. The study also determined the enhanced presence of trace elements, in addition to the higher levels of total phenolics and flavonoid contents in these plants, as a hallmark of halophytic plant characteristics.

## 2. Results and Discussion

The halophytic plants are capable of over-producing phenolics and flavonoids as part of their defense mechanisms against higher oxidative stress of the excess-salinity soil conditions in difference to their normal (non-salinity) counterparts [1]. In the current work, five halophytic plants, namely, *A. lagopoides*, *S. imbricata*, *S. cyclophylla*, *T. aphylla*, and *Z. simplex* were investigated for their phenolics, flavonoids, and mineral contents’ (iron, copper, and zinc) comparative occurrence. Moreover, based on inputs from the prevalent traditional medicinal uses of these plants in the region, the species as part of the regional plant diaspora were evaluated, for anti-microbial and anti-oxidant potentials, as well as α-amylase inhibitory effects as equivalent to the anti-diabetic activity, for comparative purposes. Various pharmacological activities have been linked to the presence of high anti-oxidants potentials of the plants [16].

### 2.1. Total Phenolics and Flavonoids Contents

The comparative quantities of the total phenolics and flavonoid contents in the halophytes, calculated as equivalents of the gallic acid and rutin-based presence, and expressed as mg of the per gram of dried extract of each plant are displayed in Table 1. Overall, these results are a supportive marker for the abundance of these types of phytochemical compounds in the halophytic plants. The total phenolics and flavonoid contents of these plants were measured in the average of 126.6 mg/g to 360 mg/g GAE, and of 20.5 mg/g to 70.5 mg/g RE of the different plants dried extracts, respectively. The quantity of total phenolics constituents was comparatively higher than their concentrations in the plants growing in non-saline conditions [25]. The results reflected the effects of environmental conditions on the halophytes’ ability to excessively biosynthesize the phenolics and flavonoids. Major differences in the total phenolics and flavonoids contents were recorded for *S. imbricata* growing in Saudi Arabia (360 mg/g GAE and 70.5 mg/g RE of the extract, respectively) to that reported for the same plant species growing in coastal areas of Karachi, Pakistan (≈8 mg/g GAE and 5 mg/g RE, respectively). A similar conclusion can also be drawn through a thorough comparison of the total phenolics contents obtained for *A. lagopoides* in comparison to the same species growing in Kolhapur, India, a non-coastal, non-sandy, non-high-salt soil region [26]. The noticeable differences in the total phenolics and flavonoids contents were also recorded for *S. imbricata* and *S. cyclophylla* which belonged to the same genus, *Salsola*, wherein the highest quantities of phenolics and flavonoids contents were found at 360 mg/g of GAE, and 70.5 mg/g of RE for the *S. imbricata*. In contrast, the lowest concentrations of phenolics and flavonoids were recorded for *S. cyclophylla* (126.6 mg/g of GAE and 20.5 mg/g of RE, respectively). Higher concentrations of phenolics and flavonoids contents were also quantified in *A. lagopoides* and *Z. simplex* that showed total phenolics contents values at 293 mg/g and 260 mg/g of GAE, and total flavonoids contents values at 54.8 mg/g, and 35.5 mg/g of RE, respectively, (Table 1).

### 2.2. Trace Elements Analysis

Trace elements form part of the cellular materials and are necessary for many biological functions. They are also important from the view-point of human nutrition and participate in several vital metabolic activities inside of the body [27]. Iron, for instance, is an essential element in hemoglobin biosynthesis [28]. A number of trace elements, including iron, copper, and zinc, have been reported in very high concentrations from the high-salinity soil of the region [29]. The trace elements analysis of these halophytes showed iron in all the plants’ samples under study, wherein the highest iron concentration was detected in *A. lagopoides* (4113 µg/kg of the plant powder), while the lowest iron concentration was found in *Z. simplex* (525 µg/kg of the plant powder). The copper, which represents the important constituent of the antioxidant superoxide dismutase enzyme and ceruloplasmin metalloproteinase [30], was also detected in all the halophytic plants under investigation. The *S. imbricata* contained the highest concentration of copper (11.1 µg/kg of the plant powder) while *S. cyclophylla* contained the lowest concentration of copper at 0.48 µg/kg (of the plant powder). All the studied halophytic plants contained zinc with concentrations ranging from 27.4 µg/kg to 40.1 µg/kg of the respective plant powders (Table 2). *A. lagopoides* contained the highest concentration of zinc at 40.1 µg/kg, while the *S. cyclophylla*, as well as the *Z. simplex*, contained the lowest levels of zinc at 27.4 µg/kg of the respective plant powders (Table 2). The copper and zinc contents of the plants, seemingly, play important roles in the plants’ biological activities. An earlier study reported that zinc possesses vital functions to perform in the secretion of insulin hormone, and is also involved in increasing the sensitivity of the tissues to insulin action. In addition, zinc has anti-diabetic, and anti-oxidant activities also [31].

### 2.3. Anti-Oxidant Activity

The anti-oxidant potentials of the halophytes were assayed by the DPPH radicals’ inhibition capacity method [32]. The highest DPPH radicals scavenging capacity was observed in the extract of *A. lagopoides* with IC_50_ value at 94.42 ± 4.19 µg/mL (Table 3). Ascorbic acid, the referral standard, showed the highest DPPH radicals’ inhibition capacity percentage at all the measured concentrations, wherein the maximum inhibition of 94.00 ± 0.26% was observed at 1000 µg/mL concentration (IC_50_ 60.49 ± 3.73 µg/mL). The plants, *Z. simplex, S. cyclophylla*, *A. lagopoides*, *T. aphylla*, and *S. imbricata* showed their maximum DPPH radicals’ inhibition activity of 90.30 ± 0.35% (IC_50_ 65.67 ± 1.49 µg/mL), 93.28 ± 0.18% (IC_50_ 76.78 ± 2.12 µg/mL), 91.04 ± 0.12% (IC_50_ 94.42 ± 4.19 µg/mL), 90.30 ± 0.39% (IC_50_ 71.01 ± 1.93 µg/mL), and 91.04 ± 0.12% (IC_50_ 66.34 ± 2.28 µg/mL), respectively, at the same, 1000 µg/mL, concentrations. Among all the studied plants, *S. cyclophylla* at 10 µg/mL extract’s concentration showed significantly increased DPPH radicals’ inhibition percentage as compared to the *Z. simplex, A. lagopoides*, *T. aphylla*, and *S. imbricata* extracts. Similarly, at 25 µg/mL, and 50 µg/mL extracts’ concentrations, the *S. cyclophylla* showed significantly increased DPPH radicals’ inhibitions as compared to all the other plants’ extracts. No significant inhibitory differences were observed among all the studied plants at 250 µg/mL concentrations of the extracts, while at 500 mg/mL concentrations, a significant increase in the inhibitory activity of *S. cyclophylla* was observed, which showed the highest inhibitory capacity compared to all other plants studied (Table 3). All the plants also scavenged the DPPH free radicals with a higher IC_50_ value which is attributable to the chemical nature of the phytoconstituents present in these plants, and they may not be the phenolics and flavonoids structural types.

The iron metal ions chelating activity of the plant extracts were compared to the di sodium-EDTA (Ethylene Diamine Tetra Acetic-Acid) as the referral standard. The results showed that *S. imbricata* extract chelated about 46.12% of the ferrous ions at the 1.25 mg/mL of minimum concentration, while at the same time, the intermediate levels of iron-chelating activities were recorded for the extracts, at the same concentration, for *Z. simplex*, *A. lagopoides,* and *T. aphylla,* which chelated the ferrous ions by 31.92%, 26.09%, and 25.47%, respectively. The *S. cyclophylla* exhibited the lowest chelating activity (19.23%) among all the tested plants’ extracts. The results revealed that these particular plants, and halophytes in general, comparatively possessed moderate to high levels of anti-oxidant behaviors than the halophytic and non-halophytic plants of same genera found in non-salinity conditions [25,26] which has been manifested through multiple channels of anti-oxidant activity testings.

### 2.4. The α-Amylase Inhibitory Activity

The α-amylase inhibitory activity is a rapid, reproducible, and indirect method for screening the anti-diabetic potential of plant extracts and their pure constituents. The halophytic nature of the plants under investigation, and the abundances of phenolics, flavonoids, and trace elements contents in their extracts, prompted to examine their potential anti-diabetic effects. The obtained anti-amylase activity data (Table 4) indicated that these halophytic plants may yield a prospective natural product-based template for an anti-diabetic drug lead. The *Z. simplex* extract demonstrated the highest capacity to inhibit α-amylase enzyme at IC_50_ value of 60.43 ± 8.29 µg/mL with significant differences in comparison to the referral standard, acarbose (IC_50_ value 40.54 ± 1.65 µg/mL). Similarly, all the other plants’ extracts demonstrated α-amylase inhibitory activity (Table 4) with IC_50_ values of 49.24 ± 1.88, 56.60 ± 8.46, 50.58 ± 2.25, and 44.66 ± 1.90 µg/mL for *S. cyclophylla, A. lagopoides, T. aphylla,* and *S. imbricata* plants extracts, respectively. The results of α-amylase based anti-diabetic activity also revealed a positive relationship between total phenolics and total flavonoids contents of the plants, and their maximum α-amylase inhibitory activity was found in *S. imbricata* extract which has the highest phenolics and flavonoids contents (Table 1). The *S. cyclophylla* possessed the lowest concentrations of phenolics and flavonoids contents in its extract, and the plant’s extract also showed the lowest α-amylase inhibitory capacity. The observation that *S. imbricata* extract possessed the highest α-amylase inhibitory capacity also corroborated its traditional use as an anti-diabetic concoction. The finding also suggested that it can be a good candidate for at least as a crude drug, and a candidate for further investigations towards developing an anti-diabetic drug.

### 2.5. Anti-Microbial Activity

The anti-microbial activity testings of the plant extracts were performed against broad-spectrum microorganisms which included Gram-positive, and Gram-negative bacteria, and *Candida albicans* fungal strain. The inhibition zone diameter (IZD) displayed by the plants’ extracts as compared to the standard antibiotics were recorded. The minimum inhibitory concentration (MIC) of the plants’ extracts against particular microorganisms, selected according to the results obtained from the IZD assays, were also determined. Most of the extracts were either inactive or had weak activity against the microorganisms, i.e., the extracts obtained from *S. imbricata* and *S. cyclophylla* exhibited weak antimicrobial activity with IZD values of 11 and 8 mm, respectively, against *S. aureus* as compared to the standard antibiotic drug. The weak anti-microbial effects were also observed for the *A. lagopoides* plant’s extract with IZD values of 9 mm and 8 mm against *Staphylococcus aureus,* and *Escherichia coli,* respectively. Additionally, the *A. lagopoides* extract was also inactive against all other tested microorganisms. The anti-microbial activity observations revealed moderate effects of *Z. simplex* and *T. aphylla* aq. ethanolic extracts against *Staphylococcus aureus* which showed equal IZD values of 13 mm, and MIC values at 125 µg/mL, and 112.5 µg/mL, respectively. The extract of *T. aphylla* moderately inhibited the growth of *E. coli*, *Enterococcus faecalis*, *Klebsiella pneumonia*, *Pseudomonas aeruginosa*, Methicillin resistance *Staphylococcus aureus* (MRSA), and the *C. albicans* with IZD values ranging from 12 mm to 15 mm areas, except for the *Proteus mirabilis* where it was inactive. Additionally, the MIC measured for *T. aphylla* extract against *E. coli*, *E. faecalis*, MRSA, and *C. albicans* were ranging between 82 and 95 µg/mL of the extract. The strongest anti-microbial activity was recorded for the extract of *Z. simplex* against MRSA with an IZD area of 33 mm, and a MIC value of 50 µg/mL. The results in Table 5 summarized the anti-microbial activities of the plants extracts, of which the *T. aphylla* was the most wide-spectrum active plant, followed by the *Z. simplex*. The results indicated that the anti-microbial potential of *Z. simplex,* which showed 33 mm IZD against one of the most widely-resisted microorganisms, i.e., MRSA, may evolve as a probable candidate to cope with the infections caused by the MRSA to humans and cattle alike [33,34]. The plant’s extract may also yield an anti-microbial lead template in the future upon further investigation. The anti-microbial potential of *T. aphylla* was most wide-spread and showed resistance against all tested microorganisms, except the *P. mirabilis*.

## 3. Materials and Methods

### 3.1. Plants Materials, and Extraction Procedure

All the plants were collected in November 2019 from the high salted arid areas in the Qassim region of central Saudi Arabia. The plant materials were identified as *S. imbricata, S. cyclophylla, T. aphylla, Z. simplex,* and *A. lagopoides* by Dr. Abdulrahman Al-Soeer, Plant Taxonomy Department, College of Agriculture and Veterinary Medicine, Qassim University. For each plant, 20 samples of aerial parts were collected, dried in the shade at room temperature (RT, 24 ± 2 °C), combined, and grinded to a coarse powder by a mechanical grinder. A weighed quantity, 500 gm of each dried plant powder were placed in large flasks (5 L volume) and were extracted with 1.5 L of 5%-aqueous ethanol at RT (24 ± 2 °C) under constant shaking for overnight over a vibrating shaker. The solvents were collected by filtrations, concentrated on a rotatory vacuum evaporator under reduced pressure, and <40 °C temperature. The dried extracts were stored under −20 °C in a deep freezer for further processing.

### 3.2. Total Phenolics Contents

The total phenolics contents (TPC) of all the plant extracts’ were determined as Gallic Acid Equivalents (GAE) using Folin-Ciocalteu reagent by the method described in the literature [8]. Concisely, Folin Ciocalteu reagent (diluted 1:10, *v/v*, distilled water, 2.5 mL) was added to the extract in a concentration of 1 mg/mL (1 mL), and saturated solution of sodium carbonate (2 mL) was added and well mixed. The mixture was shaken vigorously and set aside for 30 min at 40 °C water-bath protected from light. The absorbance of the developed blue color was measured at 765 nm by UV-VIS Spectrophotometer (Model V-630, JASCO Co., Tokyo, Japan). The total phenolics contents of the extracts were expressed as mg/g of the dried plant extracts’ equivalent to the Gallic Acid using the generated Gallic Acid calibration curve. All the results were obtained as an average of three independent measurements, and their standard deviations were calculated.

### 3.3. Total Flavonoids Contents

All the plants’ extracts were assayed for their flavonoids contents as Rutin Equivalents (RE) in mg/g of the dried plant extract using the method described by Ordonez [35]. Concisely, 1 mL of an extract in a 1 mg/mL concentration was mixed with 1 mL of aluminum chloride (2% in ethanol), and the mixture was shaken vigorously, and kept at RT (24 ± 2 °C) for 1 h, followed by measurement of the absorbance at 440 nm using a UV-VIS Spectrophotometer (V-630, JASCO Spectrophotometer). The flavonoids contents of the extracts were calculated from the generated calibration curve of rutin as mg/g of the dried extract. All the results were obtained as an average of three independent measurements, and standard deviations were calculated [36].

### 3.4. Trace Element Analysis

The trace elements, i.e., Fe, Cu, and Zn of the halophytic plants, were determined from the dried plant’s powder, as reported by Johnsson [37]. The plants were dried at 70 °C for two days and sifted by a stainless-steel mill under 5 mm pore size. The dried materials (0.5 gm, each plant separately) were digested using a solution of concentrated HNO_3_, HCIO_4_, and H_2_SO_4_ (7:2:l) [37]. The trace elements were measured by ICP-OES (Model iCAP 7400 Duo, serial IC 74DC144208, China) instrument.

### 3.5. Estimation of Anti-Oxidant Activity

The anti-oxidant potentials of the halophytic plants’ extracts’ samples were determined by estimating the iron chelating activity using ferrozine, and radicals scavenging activity through the DPPH (2,2-Diphenyl-1-picrylhydrazyl) assays [38,39]. A volume of 500 μL of DPPH (0.44 mg DPPH was dissolved in 1000 mL of 5% aqueous ethanol), and mixed with 500 μL of different concentrations of the plant extracts separately (1000 to 10 μg/mL). Similarly, blank and positive control mixtures were prepared [39]. The mixtures were then heated at 45 °C in a water-bath for 20 min, and the absorbance was recorded at 517 nm by the available UV-VIS spectrophotometer. The experimental values are expressed as the mean ± Standard Error Mean (SEM), and all the experiments were carried out in triplicates (*n* = 3). The inhibition, which reflected the radical scavenging activity, was calculated using the following equation:

(1) $$Inhibition\;\%=\left[\frac{Aa-Ad}{Aa}\right]\times 100$$

where *Aa* is the absorbance of the control, *Ad* is the absorbance of the extract.

For estimating the iron-metal chelating activity, 1 mL of each plant’s extract (varying concentrations), 50 µL of FeCl_2_ (2 mM), 0.2 mL of aqueous ferrozine solution (5 mM) were mixed and shaken for 10 min. Absorbance of all the extracts and standard was measured at 562 nm. The percent inhibition of the formed ferrozine–iron complex was calculated according to the Equation (1) [39].

### 3.6. α-Amylase Enzyme Inhibition Assay

The α-amylase enzyme inhibition potentials of the extracts were carried out according to the reported method [40]. A volume of 500 μL acarbose or halophytic plant’s sample at different concentrations were incubated with 500 μL of 0.5 mg/mL α-amylase in 0.2 mM phosphate buffer (pH 6.9) at 25 °C for 10 min, followed by the addition of 1% starch solution (500 μL) in 0.02 M sodium phosphate buffer (pH 6.9), and the mixture was incubated for 10 min at 25 °C. The dinitro-α-salicylic acid (DNS) coloring reagent (1 mL) was added, and the mixture was boiled for 5 min, cooled to RT (24 ± 2 °C), followed by the addition of 10 mL of distilled water to dilute the reaction mixture. The absorbance was recorded at 540 nm. A blank control was prepared using a similar procedure with solvent in the absence of an extract sample. The experimental value is expressed as mean ± Standard Error Mean (SEM), and all experiments were carried in triplicates (*n* = 3). The percentages of α-amylase enzyme inhibition were calculated using the following formula:

(2) $$\alpha -amylase\;enzyme\;inhibition\;\%=\left[\frac{Ac-Ae}{Ac}\right]\times 100$$

where *Ac* is the absorbance of the control, *Ae* is the absorbance of the extract.

### 3.7. Antimicrobial Activity

#### Microorganisms

*Staphylococcus aureus* ATCC 25923, Methicillin resistance *Staphylococcus aureus* (MRSA) ATCC 43300, *Enterococcus faecalis* ATCC 29212, *Escherichia coli* ATCC 35218, *Pseudomonas aeruginosa* ATCC 9027, *Proteus mirabilis* ATCC 29906, *Klebsiella pneumonia* ATCC 27736, and *Candida albicans* ATCC 10231 were used for anti-microbial testings of the plants’ extracts.

### 3.8. Determination of Anti-Microbial Activity Using the Agar Diffusion Method

The agar diffusion anti-microbial growth test method was used for estimating the anti-microbial activity of *S. imbricata, S. cyclophylla, Z. simplex*, *A. lagopoides,* and *T. aphylla* extracts. The required number of wells were cut on the agar plate using the back of a sterile glass dropper (6 mm diameter) and labeled. The microbial suspensions were prepared by taking a sample from the culture and mixing it with Muller Hinton Broth (MHB). The turbidity/growth corresponding to a 0.5 McFarland was used as standard. The Mueller Hinton Agar (MHA) plates were inoculated with the culture of each microbial suspension by spreading it evenly using sterile cotton swabs. For the fungal culture, SDA (Sabouraud Dextrose Agar) was used. Clotrimazole was used as the control for the yeast strain. The extracts were dissolved using DMSO to get the concentration of 50 mg/mL, followed by the addition of 100 μL of each extract through a micropipette into the agar wells. Gentamicin (10 μg/disc) was used as a positive control for Gram-positive bacteria, while Ciprofloxacin (5 μg/disc), and Oxacillin (1 μg/disc) were the positive control for Gram-negative bacteria. A negative control, DMSO solution of 100 μL, was poured into the wells. The plates were incubated at 37 ± 1 °C for 24–48 h and examined for anti-microbial activity [41].

### 3.9. Determination of Minimum Inhibitory Concentrations

The two-fold broth dilution method [4] was used to determine the minimum inhibitory concentration (MIC) of the extracts. 900 μL of bacterial suspension (cultured in Mueller Hinton Broth supplemented with Tween-80) was added to 100 μL of each of the extracts from the serially diluted concentration range (50, 25, 12.5, 6.25, 3.12, 1.56, 0.78, 0.39, 0.195, 0.097, 0.048 mg/mL). The mixtures were incubated at 37 ± 1 °C for 24 h, and observed bacterial growths were determined. The lowest concentration showed no turbidity, and that was considered the MIC value.

### 3.10. Statistical Analyses

Data are expressed as the mean ± standard error of the mean (SEM). The differences between groups were analyzed using one-way ANOVA followed by posthoc test using Tukey’s multi-group comparisons on GraphPadPrism 8.0.2, San Diego, CA, USA. The data were considered significant if *p* < 0.05 was obtained [42]. The superscripts used to describe the significance among the groups in the tables were obtained using Minitab 19.1. The data were normalized, and the half-maximal inhibitory concentrations (IC_50_) were obtained using a GraphPadPrism Software.

## 4. Conclusions

The higher phenolics and flavonoid contents, an abundance of iron, copper, and zinc as trace elements, together with the comparatively enhanced anti-oxidant, and anti-diabetic activity that was measured as an expression of the α-amylase inhibitory potential, established the usefulness of the five commonly encountered halophytic plants, *A. lagopoides*, *S. cyclophylla*, *S. imbricata*, *T. aphylla*, and *Z. simplex* of the region as part of the traditional medicinal chest forming the plants’ resources wealth for the population. The strong anti-oxidant potentials and the α-amylase inhibitory activity confirmed the traditional uses of these plants as sugar-control herbs, and the use of *T. aphylla* in wound healing as an anti-infection. The better phenolics and flavonoids phytoconstituents concentrations and bioactivities suggested that the plants, *S. imbricata*, and *A. lagopoides,* upon further investigations, may provide the active constituents for anti-diabetic biological activity, while the *T. aphylla* could yield the anti-microbial agent’s lead molecule, or a molecular template for further development. The *Z. simplex,* active against MRSA may throw up the MRSA-inhibiting active constituent(s). Besides, these plants may also serve as potential nutraceuticals, as food-supplements, and food for drought conditions for humans and cattle alike. The plants are of immense health value in line with their traditional and folk-lore’s medicinal-use reputation, which is also supported by their concurrent investigations, especially for the α-amylase inhibitory activity potential which is an indication of the postprandial blood glucose level reduction capacity in diabetic conditions.

## Figures and Tables

**Table 1:** Phenolics and flavonoids contents’ concentrations of the halophytes *.

Plant	TPC (mg/g)	TFC (mg/g)
*Salsola imbricata*	360.0 ± 2.01	70.5 ± 0.88
*Salsola cyclophylla*	126.6 ± 0.81	20.5 ± 1.02
*Tamarix aphylla*	159.9 ± 1.8	30.5 ± 0.96
*Zygophyllum simplex*	260.1 ± 0.94	35.5 ± 0.61
*Aeluropus lagopoides*	293.3 ± 1.60	54.8 ± 0.88

* Values are expressed as mean ± SEM; TPC, Total phenolics contents; TFC, Total flavonoids contents.

**Table 2:** Trace elements analysis of the halophytes.

Trace Element	Halophytic Plant				
	*S. cyclophylla*	*S. imbricata*	*T. aphylla*	*A. lagopoides*	*Z. simplex*
Iron (Fe) µg/kg	600	865	1474	4113	525
Copper (Cu) µg/kg	0.48	11.1	1.92	7.01	2.64
Zinc (Zn) µg/kg	27.4	35.0	35.2	40.1	27.4

**Table 3:** Halophytic plants extract’s DPPH radicals’ inhibitions.

Conc. (µg/mL)	Standard	Halophytic Plants				
	Ascorbic Acid	*Z. simplex*	*S. cyclophylla*	*A. lagopoides*	*T. aphylla*	*S. imbricata*
10	40.31 ± 0.40 ^A^	29.85 ± 0.36 ^D^	39.55 ± 0.35 ^AB^	37.31 ± 0.0.28 ^C^	38.06 ± 0.32 ^BC^	37.31 ± 0.15 ^C^
25	52.22 ± 0.16 ^A^	38.06 ± 0.24 ^F^	48.51 ± 0.22 ^B^	42.54 ± 0.15 ^E^	44.03 ± 0.21 ^D^	45.52 ± 0.26 ^C^
50	56.70 ± 0.12 ^A^	52.24 ± 0.18 ^C^	55.22 ± 0.17 ^B^	50.75 ± 0.42 ^D^	50.00 ± 0.23 ^D^	52.24 ± 0.19 ^C^
100	85.10 ± 0.08 ^A^	71.64 ± 2.52 ^D^	73.88 ± 0.24 ^CD^	64.18 ± 0.19 ^E^	77.61 ± 0.17 ^BC^	79.10 ± 0.28 ^B^
250	86.60 ± 0.15 ^A^	86.57 ± 0.12 ^A^	85.82 ± 0.16 ^A^	86.57 ± 0.27 ^A^	86.57 ± 0.19 ^A^	86.57 ± 0.27 ^A^
500	92.50 ± 0.52 ^A^	89.50 ± 0.44 ^B^	90.30 ± 0.39 ^B^	83.58 ± 0.40 ^C^	89.55 ± 0.32 ^B^	89.55 ± 0.05 ^B^
1000	94.00 ± 0.26 ^A^	90.30 ± 0.35 ^B^	93.28 ± 0.18 ^A^	91.04 ± 0.12 ^B^	90.30 ± 0.39 ^B^	91.04 ± 0.12 ^B^
IC_50_ ± SEM (95% CI IC_50_ range)	60.49 ± 3.73 (53.19 to 68.78)	65.67 ± 1.49 (62.62 to 68.78)	76.78 ± 2.12 (72.48 to 81.38)	94.42 ± 4.19 (86.12 to 103.7)	71.01 ± 1.93 (67.13 to 75.14)	66.34 ± 2.28 (61.78 to 71.25)

Values are expressed as mean ± SEM. The experiments were carried out in triplicates. Statistical significance was performed using one-way ANOVA (*p* < 0.0001), followed by a posthoc test. The mean values that do not share a letter (A–F) for the relevant concentration row (10–1000 µg/mL) are significantly different (*p* < 0.01) found using Tukey’s multi-group comparisons. For example, at concentration 10 µg/mL, all the groups (represented by letters B–D), except *S. cyclophylla* (represented by letter A), are significantly different compared to the ascorbic acid (represented by letter A); similarly, *S. cyclophylla* extract value (represented by letters A and B) is significantly different as compared to all the other groups (represented by letters C–D) except for the ascorbic acid (represented by letter A), and the *T. aphylla* plant extract’s value (represented by letter B).

**Table 4:** α-Amylase inhibitory activity of the halophytes *.

Conc. (µg/mL)	Acarbose	*Z. simplex*	*S. cyclophylla*	*A. lagopoides*	*T. aphylla*	*S. imbricata*
10	36.92 ± 0.44 ^A^	31.54±0.52 ^BC^	30.00 ± 0.68 ^C^	33.08 ± 0.43 ^AB^	31.54±0.33 ^BC^	30.77 ± 0.28 ^C^
25	46.15 ± 0.52 ^A^	47.69 ± 0.48 ^A^	38.46 ± 0.28 ^B^	38.46 ± 0.66 ^B^	40.77 ± 0.58 ^B^	46.15 ± 0.46 ^A^
50	76.92 ± 0.23 ^A^	70.00 ± 0.45 ^B^	66.15 ± 0.40 ^C^	69.23 ± 0.32 ^B^	66.92 ± 0.42 ^C^	69.23 ± 0.46 ^B^
100	84.62 ± 0.21 ^A^	71.54 ± 1.15 ^C^	77.69 ± 0.56 ^B^	71.54 ± 0.52 ^C^	77.69 ± 0.56 ^B^	77.69 ± 0.53 ^B^
250	89.23 ± 0.29 ^A^	73.85 ± 0.49 ^B^	89.23 ± 0.29 ^A^	69.23 ± 0.62 ^C^	89.23 ± 0.17 ^A^	89.23 ± 0.44 ^A^
500	91.54 ± 0.29 ^A^	82.31 ± 0.44 ^C^	90.00 ± 0.18 ^AB^	82.31 ± 0.17 ^C^	89.23 ± 0.65 ^B^	90.00 ± 0.21 ^AB^
1000	93.85 ± 0.21 ^A^	93.46±0.17 ^AB^	92.31 ± 0.40 ^B^	86.92 ± 0.29 ^C^	93.46 ± 0.28 ^AB^	93.48 ± 0.39 ^AB^
IC_50_±SEM (95% CI IC_50_ range)	40.54 ± 1.65 (37.26 to 44.25)	60.43 ± 8.29 (45.34 to 81.18)	49.24 ± 1.88 (45.44 to 53.47)	56.60 ± 8.46 (41.25 to 80.22)	50.58 ± 2.25 (46.06 to 55.69)	44.66 ± 1.90 (40.83 to 48.92)

* Values are expressed as mean ± SEM. The experiments were carried out in triplicates. Statistical significance was performed using one-way ANOVA (*p* < 0.0001), followed by a posthoc test. The means that do not share a letter (A–C) for the relevant concentration row (10–1000 µg/mL) are significantly different (*p* < 0.01) found using Tukey’s multi-group comparisons. For example, at concentration 10 µg/mL, all the groups (represented by letters B and C), except *A. lagopoides* (represented by letter A), are significantly different as compared to acarbose (represented by letter A); similarly, *A. lagopoides* plant’s extract value (represented by letters A and B) is significantly different as compared to *S. cyclophylla* and *S. imbricata* plants’ extracts values (represented by letters C).

**Table 5:** Anti-microbial activity of halophytic plants’ extracts *.

Test Organism	*Z. simplex*	*S. cyclophylla*	*A. lagopoides*	*T. aphylla*	*S. imbricata*	Positive Control
*S. aureus*	13 mm	8 mm	9 mm	13 mm	11 mm	20 mm
*P. mirabilis*	-	-	-	-	-	-
*E. coli*	-	-	8 mm	14 mm	-	35 mm
*E. faecalis*	-	-	-	14 mm	-	8 mm
*K. pneumonia*	-	-	-	12 mm	-	26 mm
*P. aeruginosa*	12 mm	-	-	12 mm	-	27 mm
*C. albicans*	-	-	-	14 mm	-	16 mm
MRSA	33 mm	-	-	15 mm	-	11 mm

*—no activity detected.
